# Together Intra-Tumor Hypoxia and Macrophagic Immunity Are Driven Worst Outcome in Pediatric High-Grade Osteosarcomas

**DOI:** 10.3390/cancers14061482

**Published:** 2022-03-14

**Authors:** Charlotte Nazon, Marina Pierrevelcin, Thibault Willaume, Benoît Lhermitte, Noelle Weingertner, Antonio Di Marco, Laurent Bund, Florence Vincent, Guillaume Bierry, Anne Gomez-Brouchet, Françoise Redini, Nathalie Gaspar, Monique Dontenwill, Natacha Entz-Werle

**Affiliations:** 1 Pediatric Onco-Hematology Unit, University Hospital of Strasbourg, 1 Avenue Molière, CEDEX, 67098 Strasbourg, France; charlotte.nazon@chru-strasbourg.fr (C.N.); florence.vincent@chru-strasbourg.fr (F.V.); 2 CNRS UMR 7021, Laboratory of Bioimaging and Pathologies, Faculty of Pharmacy, 74 Route du Rhin, 67401 Illkirch, France; marina.pierrevelcin@etu.unistra.fr (M.P.); benoit.lhermitte@chru-strasbourg.fr (B.L.); monique.dontenwill@unistra.fr (M.D.); 3 Radiology Department, University Hospital of Strasbourg, 1 Avenue Molière, CEDEX, 67098 Strasbourg, France; thibault.willaume@chru-strasbourg.fr (T.W.); guillaume.bierry@chru-strasbourg.fr (G.B.); 4 Pathology Department, University Hospital of Strasbourg, 1 Avenue Molière, CEDEX, 67098 Strasbourg, France; noelle.weingertner@chru-strasbourg.fr; 5 Department of Orthopedic Surgery and Traumatology, University Hospital of Strasbourg, 1 Avenue Molière, CEDEX, 67098 Strasbourg, France; antonio.dimarco@chru-strasbourg.fr; 6 Department of Pediatric Surgery, University Hospital of Strasbourg, 1 Avenue Molière, CEDEX, 67098 Strasbourg, France; laurent.bund@chru-strasbourg.fr; 7 Department of Pathology, University Hospital of Toulouse, 1 Avenue Irène Joliot Curie, 31100 Toulouse, France; gomez.anne@chu-toulouse.fr; 8 INSERM UMR1238, PHY-OS, Bone Sarcomas and Remodeling of Calcified Tissues, Nantes University, 44000 Nantes, France; francoise.redini@univ-nantes.fr; 9 Department of Oncology for Children and Adolescents, Gustave Roussy, 94805 Villejuif, France; nathalie.gaspar@gustaveroussy.fr; 10 INSERM U1015, Gustave Roussy, University of Paris-Saclay, 94805 Villejuif, France; 11 University of Paris-Saclay, 91400 Orsay, France

**Keywords:** osteosarcoma, pediatric, biomarkers, hypoxia, macrophages, magnetic resonance imaging

## Abstract

**Simple Summary:**

Radiological and immunohistochemical data were correlated with the outcome in a retrospective monocentric cohort of 30 pediatric osteosarcomas (OTS). A necrotic volume of more than 50 cm^3^ at diagnosis was significantly linked to a worse overall survival (OS). Regarding immunohistochemical analyses, an overexpression of hypoxic markers, such as HIF-1α and anhydrase carbonic IX (CAIX), was significantly linked to a worse OS, while pS6-RP hyperexpression was correlated with a better survival. We also featured that CD68 positive cells, representative of macrophagic M1 polarization, were mostly associated with HIF-1α and CAIX hyperexpressions and that M2-like polarization, mostly related to CD163 positivity, was correlated to mTor activation. These findings, involving clinical, radiological and biology data, allowed us to hypothesize a dual signature association ready to use routinely in future protocols.

**Abstract:**

Background: Osteosarcomas (OTS) represent the most common primary bone cancer diagnosed in adolescents and young adults. Despite remarkable advances, there are no objective molecular or imaging markers able to predict an OTS outcome at diagnosis. Focusing on biomarkers contributing broadly to treatment resistance, we examine the interplay between the tumor-associated macrophages and intra-tumor hypoxia. Methods: Radiological and immunohistochemical (IHC) data were correlated with the outcome in a retrospective and monocentric cohort of 30 pediatric OTS. We studied hypoxic (pS6, phospho-mTor, HIF-1α and carbonic anhydrase IX (CAIX)) and macrophagic (CD68 and CD163) biomarkers. Results: The imaging analyses were based on MRI manual volumetric measures on axial post-contrast T1 weighted images, where, for each tumor, we determined the necrotic volume and its ratio to the entire tumor volume. When they were above 50 cm^3^ and 20%, respectively, they correlated with a worse overall survival (*p* = 0.0072 and *p* = 0.0136, respectively) and event-free survival (*p* = 0.0059 and *p* = 0.0143, respectively). IHC assessments enable a significant statistical link between HIF-1α/CAIX hyper-expressions, CD68+ cells and a worse outcome, whereas activation of mTor pathway was linked to a better survival rate and CD163+ cells. Conclusions: This study evidenced the links between hypoxia and immunity in OTS, as their poor outcome may be related to a larger necrotic volume on diagnostic MRI and, in biopsies, to a specific IHC profile.

## 1. Introduction

Even though osteosarcoma (OTS) is rare, with only 3 cases per million inhabitants a year [1,2], it is the most common primary bone cancer. Sixty percent of those tumors occur in children ranging from 10 to 20 years old with a male predominance [1]. The introduction of multi-agent chemotherapy several decades ago improved 5-year event-free survival (EFS) from less than 20% to 70% in localized high-grade OTS. Since the 1980s, the 5-year overall survival rates (OS) have remained stable for all OTS independently from treatment protocol: around 80% for localized osteosarcoma and 20% for metastatic disease with poor response to chemotherapy [1,3,4,5,6,7,8].

The management of OTS patients lacks theranostic markers at diagnosis predicting the response to chemotherapy and outcome. The only valuable and prognostic marker available for now is the estimated Rosen grading on the histological analysis of the primary tumor’s resection. The evaluation of residual viable cells is set up at 10% and is splitting patients in good responders (GR) and poor responders (PR) to chemotherapy after 4 months of treatment. The second prognostic marker is the metastatic status at diagnosis. Nevertheless, every patient is biopsied to confirm the diagnosis of osteosarcoma, allowing oncologists to have available tumor tissues to explore and determine new innovative predictive biomarkers.

OTS, similarly, to most solid cancers, are necrotic tumors [1]. To our knowledge and to date, no correlation has been published between hypoxia and the percentage of necrosis in those cancers. The tumor microenvironment has a clear influence on the growth of malignant cells and affects the expression of genes responsible for proliferation, migration, metabolism and viability of tumor cells [9,10,11,12]. Hypoxia, by developing an apoptotic resistance and a decrease of DNA reparation capacity, leads to therapeutic resistances [13,14,15,16,17]. Knowing that, we wonder about the impact of hypoxia in OTS. Formerly, numerous studies on pediatric OTS have implied hypoxic biomarkers, such as VEGF/VEGFR (Vascular Endothelial Growth Factor/Receptor), mTor (mechanistic Target of rapamycin) or angiopoietin [18,19,20,21,22]. Gradually, other preclinical studies, using cellular, animal models or tumors’ collections have focused on markers, such as Hypoxia Inducible Factor-1 alpha (HIF-1α) or Carbonic Anhydrase IX (CAIX). They evidenced significant links between their overexpression and a worse outcome. Intra-tumor hypoxia mechanism is based on the upstream activation of mTor/HIF-1α by RAS/MAPK and Pi3K/AKT pathways. After his own phosphorylation, phospho-mTor (pmTor) activates other nuclear transcription factors, such as HIFs via pS6K phosphorylation. Following hypoxic stress, a direct activation of HIF-1α can also occur via the Von Hippel Lindau (VHL) protein enhancing its stabilization. In that respect, HIF-1α then induces the transcription of genes, which stimulate angiogenesis, erythropoiesis and anaerobic glycolysis. Therefore, it compensates the rarity of oxygen in the tumor by using different pathways of survival. Thus, HIF-1α, CAIX, pS6-RP and pmTor might constitute an accurate hypoxic signature to study in OTS.

Apart from extracellular features, the OTS micro-environment also presents an immune niche mostly composed of tumor-associated macrophages (TAMs). They interact with tumor growth mostly due to their polarization M1/M2. M1-macrophages are schematically considered as anti-tumor and pro-inflammatory effectors and M2 macrophages and osteoclasts as pro-tumor and anti-inflammatory modulators. According to previous studies, the density of M2 TAMs seems to be correlated to tumor proliferation, metastatic process and poor outcome [23,24,25,26]. The ancillary study of OS2006 protocol demonstrated that a percentage above 50% of CD163 positive (CD163+) mononuclear cells was significantly associated with better survival [27], which was challenging the concept of CD163+ M2 macrophages. Nevertheless, in the same study, the high co-expression of CD163 with c-MAF, a transcription factor associated to M2 macrophage polarization, was questioning the importance of the M1/M2 balance in the response to zoledronic acid targeting OTS immunity. When TAMs have a CD68 staining, OTS were associated with a slightly poorer prognosis [25,26,27], redefining the M1/M2 dichotomy based on CD163 and CD68 staining. This redefinition of anti- and pro-tumor roles of the macrophages has been correlated with intra-tumor hypoxia in other cancers [28].

Necrosis, hypoxic tumor sites and osteoclasting activity can be also macroscopically approximate through radiological exploration, such as magnetic resonance imaging (MRI). The MRI examination is recommended at OTS diagnosis using non-contrast T1 weighted (T1W), fat-saturated T2 weighted (FST2W), Short Tau Inversion Recovery (STIR) and post-contrast fat-saturated T1 weighted sequences (FST1W) [29]. It is considered as the most effective method for local extension assessment and preoperative evaluation in bone tumors [30,31]. Exploration of hypoxic regions remains challenging in imaging, but feasible with precise acquisition sequences. It requires a long time of analysis and specific software to be interpreted by radiologists. Thus, the percentage of necrotic/hypoxic regions can be measured on T1W post-contrast sequences. Basically, diagnostic necrotic areas correspond to MRI unenhanced locations and can be contoured and measured. Up to date, in OTS, only a correlation between tumor volume and the patient’s survival was evidenced [4,32]. Now, new innovative radiological approaches are also developed, such as radiomics to extract with specific data-characterized algorithms predictive features for response to neoadjuvant chemotherapies [33,34]. Nevertheless, routine, simpler and reproducible radiological approaches need to be developed to specifically explore the hypoxic zones at diagnosis and link them to clinical and histological data.

Herein, this pilot study proposed to retrospectively measure the volume and percentage of non-enhanced areas on the MRI sequences in a monocentric cohort of OTS at diagnosis. In parallel, we studied on the paired diagnostic biopsic samples the expression of hypoxic and macrophagic biomarkers by immunohistochemistry (pS6-RP, pmTor, HIF-1α, CAIX, CD68, CD163 and the presence of giant cells). We focused on pediatric patients diagnosed between 2007 and 2018 to have a minimal follow-up of 24 months after the end of treatments. Lastly, we correlated these protein and radiological results to the patients’ clinical data (response to chemotherapy, metastatic status, overall and EFS) to define MRI and hypoxic signature to use as new theranostic tools in OTS retrospective and future large cohorts of therapeutic trials.

## 2. Materials and Methods

### 2.1. Patient and Tumor Data

A retrospective analysis was performed on 30 patients diagnosed for a high-grade OTS in the pediatric onco-hematological unit of University Hospital in Strasbourg between May 2007 and October 2018. All patients were treated homogeneously in the OS2006 protocol or as per-protocol [4]. Before any treatment, all patients and/or their parents (or guardians) consented for the local research use of their tumor samples. The specimens are stored at the Centre de Ressources Biologiques in the Pathology department. Only 29 formaldehyde fixed paraffin embedded (FFPE) biopsic samples were available and used for immunohistochemistry. This study was conducted in accordance with the ethical committee approval. All patient records and information were anonymized before analysis.

After diagnostic biopsy, the treatment started with a neo-adjuvant chemotherapy, except one patient who had an upfront surgery because of an initial complex histology. Five out of 30 patients were randomized for zoledronate treatment. All patients had a complete surgical resection of the primary tumor and post-surgical chemotherapy was adapted to the histological response evaluated by pathologists on resected tumor tissues, as recommended in OS2006 protocol [4]. One patient declined the tumor surgery and kept chemotherapeutic treatment. We excluded this patient from the correlation with tumor response. Before 2015, metastatic patients were treated postoperatively independently from their tumor response with cisplatin plus doxorubicin, whereas, after 2015, they received the same pre- and post-operative chemotherapies in case of good response to neo-adjuvant treatment.

### 2.2. MRI and Its Processing

Radiological evaluations were performed on initial pre-therapeutic MRI examinations, screened retrospectively from the Picture Archiving and Consulting System (PACS). All MRI were performed on a 1.5 Tesla MRI unit. Standard MRI protocol included segmental exploration of the lesion with axial FST2W images, axial and coronal pre-contrast T1W images, axial and coronal post-contrast FST1W images (slice thickness from 3 to 5 mm, interslice gap from 2 to 4 mm). The studied parameters were, for each patient, the volumetric measure of the entire tumor, the volumetric measure of tumor necrosis defined as intra-tumor unenhanced area(s) on post-contrast sequences and the percentage of tumor necrosis obtained by dividing the volume of necrotic tumor by the entire tumor volume. Manual volumetric measures were, therefore, blindly performed by two independent medical doctors (one pediatrician and one musculoskeletal radiologist) on axial post-contrast T1W images, using the Centricity Universal Viewer software^®^ (GE Healthcare, Chicago, IL, USA). First, the tumor margins were manually delineated on all sections allowing reconstruction of a 3D-volumetric model corresponding to the entire tumor volume. Then, following the same procedure, the volume of necrotic parts in the tumor was assessed after manual segmentation of unenhanced intra tumor area(s). When post-contrast MRI examination was not available, the volume of necrotic tumor was not measurable, and the entire tumor volume was assessed on axial T1W images.

### 2.3. Immunohistochemical Analyses on FFPE Samples

Immunohistochemistry (IHC) was assessing the status of pS6-RP, pmTor, HIF-1α, CAIX, CD68, CD163 expressions and the presence of giant cells (GC) in diagnostic FFPE samples. The paraffin embedding was made with the TES99 Madite system and slides were cut with a microtome Microm HM 355S (Thermo Scientific, Waltham, MA, USA) to obtain histological sections with a 4-µm thickness. The immunohistochemical staining was performed using an automated BenchMark Ultra Ventana XT (Ventana Medical system, Inc., Tucson, AZ, USA). The dewaxing of the FFPE samples by heating (100 °C) and drying allowed an antigenic restoration. A pre-treatment using ULTRA Cell Conditioning 1 (pH 8–8.5) buffer (05424569001, Roche, Basel, Switzerland) was applied. Endogenous peroxidase activity was blocked using the CM inhibitor (Ventana, Oro Valley, AZ, USA). Incubation with each specific diluted antibody was followed by a standard signal amplification with ultraWash and the use of ultraView Universal DAB Detection kit (Ventana). We used the following primary antibodies: CD68 (clone KP1, M0814, dilution: 1/1000^e^, Dako, Glostrup, Denmark), CD163 (clone 10D6, MS-1103-S, dilution: 1/200^e^, Neomarkers, Portsmouth, NH, USA), HIF-1α (ab8366, dilution: 1/1000^e^, Abcam, Cambridge, UK), pmTor (clone 49F9, dilution: 1/100^e^, Cell Signaling, Danvers, MA, USA), pS6-RP (clone54D2, Cell Signaling, dilution: 1/100^e^) and CAIX (clone MRQ-54, dilution: 1/100^e^, Cell Marque, Rocklin, CA, USA). After antibody staining, the slides are flushed by hot soapy water, dehydrated in alcohol, and put in xylene. Then, slides are inserted in the splicers (Microm CTM6, Thermo Scientific^TM^), which fix the slat using a mounting medium (Cytoseal™ XYL) and the anti-oxidative xylene. The staining analysis was performed by an expertized pathologist. The scoring system was considering a positive sample if staining is detected in more than 5% of cells per core of 1 mm. The histological study was also evaluating the presence and the percentage of GC stained by CD163 and/or CD68. Lymphoid nodes, tonsil, SEGA (subependymoma giant cell astrocytoma), lung and colorectal cancer samples were used as positive controls for CD68, CD163, pmTor/pS6-RP, HIF-1α and CAIX, respectively.

### 2.4. Statistical Analyses

Data are summarized as the frequency and percentage for categorical variables and the median and range for continuous variables. OS was defined as the time from biopsic diagnosis to death from any cause (event) or the last follow-up (censored data). EFS was defined as the time from biopsic diagnosis to relapse, progression or death. All survival correlations and Hazard-ratio (HR) were estimated by the Kaplan–Meier method with 95% confidence intervals (CI). Receiver operator characteristics (ROC) were performed to analyze the sensitivity, specificity and find a cut-off for every studied marker. The impact of immunohistochemical markers and radiologic measures was analyzed using the Kaplan–Meier method. Univariate analyses were performed using the log-rank test. Correlations between quantitative data were assessed using the Spearman’s rank correlation coefficient. Rho is the correlation coefficient and indicates a negligible correlation when between 0 and 0.30, a low correlation when between 0.30 and 0.50 and a moderate, high and very high correlation when between 0.50 and 0.70, 0.70 and 0.90, 0.90 and 1.00, respectively. Two-sided *p*-values < 0.05 were considered statistically significant. All statistical analyses were performed using GraphPad Prism v8.4.3 software (GraphPad software, Inc., San Diego, CA, USA).

## 3. Results

### 3.1. Clinical Characteristics of the Studied Cohort

**Figure 1:**
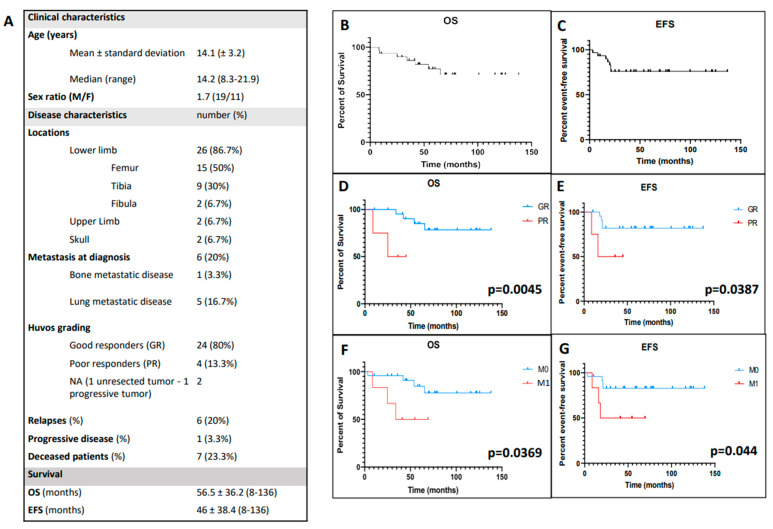
Summary of clinical and survival characteristics in our 30-patient cohort. (**A**) Table of all clinical features. (**B**,**C**) Kaplan–Meier curves reporting overall survival (OS) and event-free survival (EFS) in the cohort. (**D**–**G**) Kaplan–Meier significant correlations with clinical characteristics (**C**–**F**). The listed clinical characteristics are histological responses after chemotherapy (PR = poor responders, GR = good responders) and metastatic status at diagnosis (M0 = localized disease without metastasis, M1 = metastatic disease). *p* values are reported on the right corner of graphs and are significant when *p* < 0.05.

The demographic and clinical data are summarized in the table of Figure 1A. The 30 patients were aged from 8.3 to 21.9 years with a male predominance (19/11). They had predominantly lower limb OTS (86.7%) and six were metastatic at diagnosis (20%), with mainly lung metastases (five cases). Eighty percent were GRs after preoperative chemotherapy. One patient had an initial progressive disease and six patients relapsed with a median time of 19 months (8–21). Among those six relapsing patients, three were metastatic at diagnosis, one was a PR and one refused primary tumor surgery. Seven patients died (23.3%) including the six who relapsed. The median follow-up was 2.8 years. The population had a median OS of 56.5 months (8–138) and a median EFS of 46 months (8–138) (Figure 1B,C). OS and EFS were significantly worse for PRs (*p* = 0.0045 and *p* = 0.039, respectively) and metastatic patients (*p* = 0.037 and *p* = 0.044, respectively) (Figure 1D–G). Median time between MRI at diagnosis and evaluation of histological response was 15.8 weeks (5.3–19.8).

### 3.2. Entire Necrotic Volume (NV) and the Necrotic Percentage (NP) at Diagnosis Represent New Predictive Radiological Markers of Outcome in OTS

**Figure 2:**
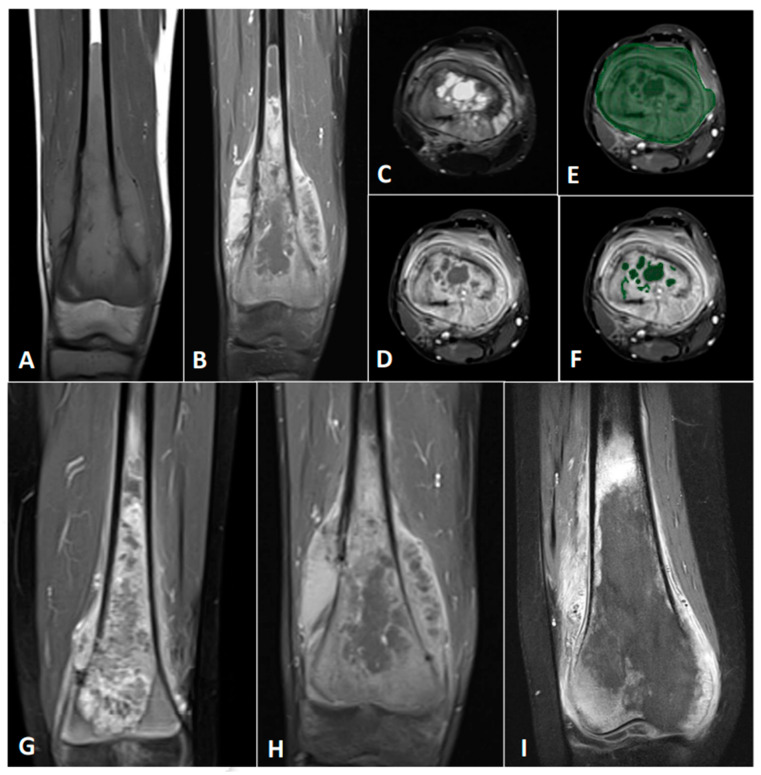
Magnetic resonance imaging (MRI) analyses. (**A**–**D**). Example of a high-grade osteosarcoma (OTS) at diagnosis in a 15-year-old patient: coronal T1 weighted image (**A**), post-contrast fat-saturated (FS) T1 weighted image (**B**), axial FS T2 weighted image (**C**) and post-contrast axial FS T1 weighted image. (**E**) Manual delineation of the tumor volume margins. (**F**) Manual delineation of intra-tumor necrotic areas. (**G**–**I**) Three different levels of necrosis in bottom line figures: coronal post-contrast FST1 weighted MRI images with a small proportion of necrosis (**G**), an average proportion of necrosis (**H**) and a high percentage of necrosis (**I**).

**Figure 3:**
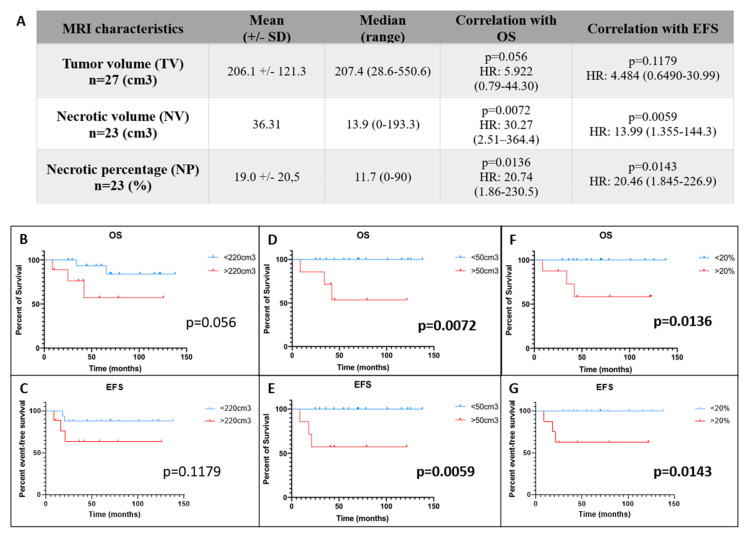
Radiological measures and their statistical correlations with survivals. (**A**) Table summarizing the MRI characteristics and the measures. (**B**) OS and (**C**) EFS distributions according to the entire tumor volume (TV) (optimal cutpoint at 220 cm^3^). (**D**,**E**) Kaplan–Meier estimates of OS and EFS according to the tumor necrotic volume (NV) (optimal cutpoint at 50 cm^3^). (**F**,**G**) Kaplan–Meier estimates of OS and EFS according to the optimal cutpoint for necrotic percentage (NP) (e.g., 20%). *p* values are reported on the right corner of the graphs, in bold when *p* < 0.05.

Three MRI out of 30 at diagnosis were uninterpretable and the tumor volume was not measurable. Seven patients did not have post-contrast T1W images and consequently the necrotic volume was not measurable for them. In Figure 2, we are showing the sequences to use for the manual delineation of the entire tumor volume and the tumor necrotic parts (e.g., Figure 2A–F) and the three levels of necrosis we could define in OTS tumors (e.g., Figure 2G–I). Based on those measures, we calculated and reported in the table of Figure 3A means and medians for the following imaging parameters: the entire tumor volume (TV), the tumor necrotic volume (NV) and the subsequent necrotic percentage (NP). Based on ROC analyses, a cut point of 220 cm^3^ for the TV on MRI was calculated. Its sensitivity was 60% and its specificity was 71.43% (area under the ROC curve (AURC): 0.666, *p* = 0.255). A cut-off for the NV of 50 cm^3^ was determined and had a sensitivity of 100% and a specificity of 78.95% (AURC: 0.8596, *p* = 0.049). For the NP, the calculated cut-off of 20% had a 100% sensitivity and a 73.68% specificity (AURC: 0.8246, *p* = 0.077). When considering the TV, a trend for a better survival was observed for patients with a volume lower than 220 cm^3^ (*p* = 0.056) (Figure 3B), but no correlation was found with EFS (Figure 3C). A NV above 50 cm^3^ and a NP above 20% were significantly linked to a worse OS (*p* = 0.0072 and *p* = 0.0136, respectively) on Figure 3D,F with a HR of 30.27 (95% CI 2.51–364.4) and 20.74 (95% CI 1.86–230.5), respectively. These bigger NVs and the paired higher NP were also significantly correlated to a worse 5-year EFS (*p* = 0.0059 and *p* = 0.0143, respectively) (Figure 3E,G) with a HR of 13.99 (95% CI 1.355–144.3) and 20.46 (95% CI 1.845–226.9), respectively.

**Figure 4:**
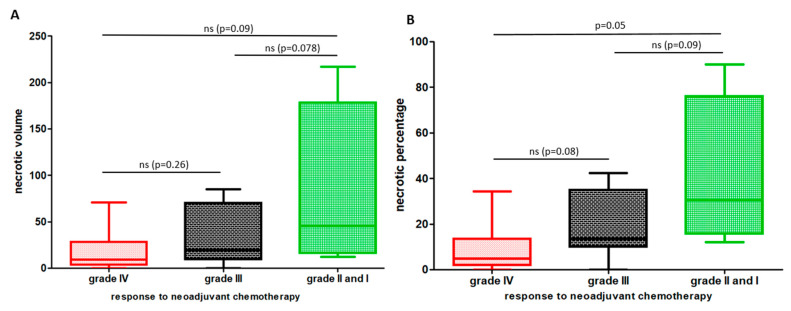
Correlations between radiological measures (NV, necrotic volume, (bar graph (**A**)) and NP, necrotic percentage (bar graph (**B**)) and Huvos grading observed on primary tumor resections. Grade IV group is represented in a red color, grade III group in black and the poor responders (grade II and I) in green. A global significancy was evidenced between these 3 groups when considering NP (*p* = 0.052) and only a minimal trend considering NV (*p* = 0.08). The statistical group-by-group analysis on the graphs (**A**,**B**) is reported above each bar plot and finds only a significant difference (*p* = 0.05) between grade IV and grade II/I Huvos groups on NP parameter. *p* values are reported on the graphs. (ns = non-significant result).

When looking to clinical data correlations, significances were only underlined globally between all Huvos grading and NP (*p* = 0.052), whereas only a minimal trend was evidenced between those different grades and NV (*p* = 0.08). Nevertheless, a clear difference is observed for those parameters between grade IV and grade II/I (Figure 4A,B). The grade II/I group is clearly reaching the worst prognostic NP with a NP mean above 20% (e.g., 36%).

### 3.3. Hypoxic and Macrophagic Immunohistochemical Biomarkers Correlate with Survivals

**Figure 5:**
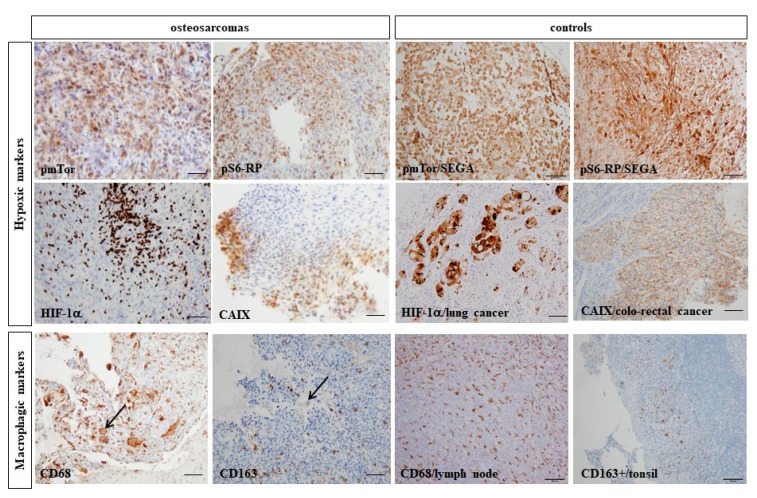
Examples of immunohistochemical staining with hypoxic markers (**upper panels**: phospho-mTor (pmTor), pS6-RP, HIF-1α and CAIX and their paired controls) and macrophagic markers (**bottom panel**: CD68 and CD163 with their paired controls). Scalebar = 20 µm, black arrows show examples of intra-tumor giant cells.

The staining results for all biomarkers are summarized in Table 1. No residual stored FFPE sample of initial biopsy was usable for one patient and the analysis could not be performed due to too small residual samples in three cases for CAIX staining, two for pmTor and one for HIF-1α, CD163 and CD68 assessments. Examples of positive immunostaining are illustrated in Figure 5.

**Figure 6:**
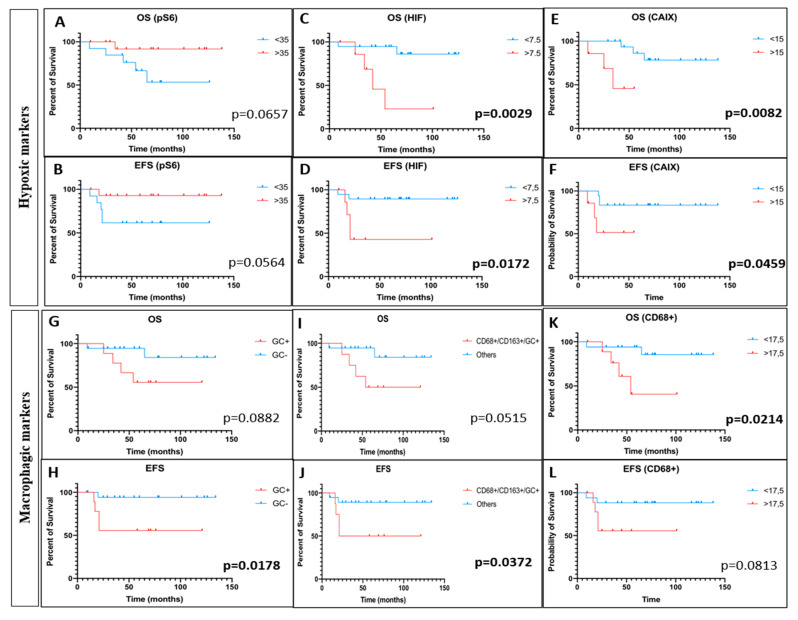
Kaplan–Meier significant correlations with immunohistological analyses of hypoxic and macrophagic biomarkers. On the upper rows, Kaplan–Meier estimates curves of OS and EFS according to the expression of pS6-RP (pS6) ((**A**,**B**), respectively), HIF-1α (HIF) (**C**,**D**), respectively) and carbonic anhydrase IX (CAIX) ((**E**,**F**), respectively). On the two last rows, Kaplan–Meier estimates of OS (**G**) and EFS (**H**) by the presence or absence of intra-tumor giant cells (GC). We stratified the population with three biomarkers associating CD68, CD163 and GC presence to a worse OS and EFS ((**I**,**J**) curves). Finally, OS and EFS distributions based on the expression of CD68 based on the cutpoint of 17.5% expression ((**K**,**L**) graphs, respectively). *p* values are reported on the right corner of the graphs, in bold when *p* < 0.05.

For hypoxic biomarkers, a high frequency of mTor activation through pS6-RP (25/29, 86.2%) and pmTor (19/27, 70.4%) hyper-expressions was evidenced, whereas HIF-1α (14/28, 50%) and CAIX (12/26, 46.1%) were less frequently expressed in OTS (Table 1). For the statistical correlations with clinical data, we considered, first, as a positive expression when more than 5% of staining cells are identified on histological slides, and as a negative response, when no expression or a staining less than 5% was observed. Using this simple cut-off, there was no correlation between pS6-RP, pmTor, HIF-1α, CAIX and survivals (data not shown). Nevertheless, ROC curves allowed finding a cutpoint for each IHC parameter in the OTS population. A cutpoint of 35% for pS6-RP expression was calculated. Its sensitivity was 83.33% and its specificity was 63.64% (AURC: 0.6705, *p* = 0.2078). A cut-off for pmTor expression of 7.5% was determined and had a sensitivity and specificity of 50% (AURC: 0.5, *p* > 0.999); it pertained 14 patients when above this limit. A trend for a worse OS and EFS was evidenced for the nine patients with <35% pS6–RP positive cells (*p* = 0.065 and *p* = 0.056, respectively) (Figure 6A,B), whereas pmTor was not correlated with any survival (OS, *p* = 0.5865 and EFS, *p* = 0.9989) (Supplemental Figure S1A,B). For HIF-1α expression, 7.5% was the determined cut-off with a sensitivity of 66.67% and a specificity of 80.95% (AURC: 0.6944, *p* = 0.153) and it was 15% for CAIX (50% sensitivity and a 78.95% specificity (AURC: 0.5395, *p* = 0.7746)). The subgroup (8/28, 28.5%) with an expression of HIF-1α over 7.5% was significantly correlated to a worse OS (*p* = 0.0029) and EFS (*p* = 0.0172) with an HR of 22.86 (95%CI 2.910–179.5) and 9.676 (95% CI 1.496–62.59), respectively (Figure 6C,D). Finally, an expression of CAIX over 15% was significantly linked to a worse OS (*p* = 0.0082) and EFS (*p* = 0.0459) with a HR of 25.34 (95% CI 2.308–278.1) and 8.357 (95% CI 1.04–67.16), respectively (Figure 6E,F). We reported this high expression in seven out of 26 patients (26.9%).

For macrophagic biomarkers, we observed a global CD68 staining in 19/28 tumors (67.8%) and a CD163 expression in 15/28 specimens (53.6%) (Table 1). When looking globally to the 5% positivity of staining in the samples, we were not able to find significant correlations except for the single parameter considering the presence of GC in the tumors. The presence of GC was concerning one third of the tumors and no obvious link was demonstrated with OS (Figure 6G, *p* = 0.0882), whereas their observation was linked with a worse EFS (*p* = 0.0178) (Figure 6H). Similarly, the only another significant correlation that was evidenced was between the eight tumor samples bearing a concomitant positive staining for CD68 and CD163 above 5%, the presence of GC and a worse OS and EFS (*p* = 0.0515 and *p* = 0.0372, respectively) (Figure 6I,J). To check correlations accurately, we performed, as in hypoxic markers, ROC analyses to determine significant cutpoints. We showed that a hyperexpression of CD68 in more than 17.5% (sensitivity 66.67%, specificity 71.43%, AURC: 0.5913, *p* = 0.5) of the sample cells was significantly linked to a worse OS (*p* = 0.0214) with an HR of 8.947 (95% CI 1.384–57.83) (Figure 6K), but not with EFS (Figure 6L). This high CD68 cell positivity was present in 10 patients out of 28 (35.7%). No statistical links were obtained for CD163 and for CD68+/CD163+ even after refining the cut-off with ROC analyses (Supplemental Figure S1C–F).

### 3.4. Hypoxic and Macrophagic Immunohistochemical Biomarkers Correlate with Survivals

In Table 1, we compared the hypoxic and macrophagic markers. For that purpose, we studied the correlation between CD68+ cells and each hypoxic marker (two by two). We found a statistically significant low correlation between CD68+ cells and pS6-RP positivity (*p* = 0.0441), and a moderate correlation between CD68 positivity and HIF-1α, pmTOR, CAIX (*p* = 0.0017, *p* = 0.0014 and *p* = 0.0084, respectively). CD163 positivity was also correlated to each hypoxic marker, and we evidenced a statistically significant moderate correlation between CD163+ cells and pS6-RP (*p* = 0.0012) and a high correlation between CD163+ cells and pmTor (*p* < 0.0001). Those results featured that macrophagic M1 type cells, schematically linked in literature to CD68 positivity, were mostly correlated with HIF-1α and CAIX markers and that M2 type cells, usually linked to CD163 positivity, were correlated with mTor activation (pS6-RP and pmTor). Herein, those data allowed us to speculate on a dual proteic signature association CD68/HIF-1α/CAIX and CD163/pS6/phospho-mTor, which is predictive of patient outcome.

Unfortunately, there was no statistically significant correlation between TV, NT, and NP cutpoints and any immunohistochemistry marker (Table 1). The only significancy that could be underlined was between the presence of GC and a higher necrotic volume (Supplemental Figure S1G–J). No correlations were observed between all these IHC markers and the metastatic status at diagnosis or the response to chemotherapy. A multivariate analysis was not feasible due to the too small size of our population.

## 4. Discussion

Over the past three decades, despite several clinical trials, the survival of patients treated for OTS has not improved. The histological evaluation of the chemotherapeutic response is too late in the journey of patient care and too approximative. In our study, four of the six patients who relapsed had an initial good histological response and only two were metastatic at diagnosis. Therefore, there is a clear need to improve our risk assessment for OTS patients at diagnosis and develop methods or assessments to estimate this risk. For this purpose, as in another trial [27], we used diagnostic biopsies to establish a signature comprising hypoxic and macrophagic biomarkers and expected correlating them to imaging parameters. We chose to retrospectively analyze a homogeneously treated cohort of OTS. This population of patients was representative of the entire OS2006-treated cohort [4,7], where we observed a male predominance, a majority of adolescents and an overall survival of 77% (e.g., 23.3% of deceased patients). The clinical characteristics (tumor site, proportion of metastatic disease, localization, etc.) were also concordant with the OS2006 results and the previous literature on OTS [8]. Surprisingly, we numbered a higher proportion of GRs to chemotherapy (80%), which can statistically bias our own study.

In our radiological data, we highlighted a significant correlation between an increased necrotic part of the tumor (NP) and a worse OS and EFS. We were also able to set up the significant cut-off of the NV over 50 cm^3^ or the NP over 20%. Surprisingly, we enable only a trend for the entire TV, which was previously considered in the OS2006 and the previous French OS94 protocol as a diagnostic predictive MRI marker [4,7,35]. One of the reasons explaining this difference is probably the lower cut-off obtained after ROC analyses than what was published in those previous cohorts. The standard for monitoring the solid tumor size is outlined in the guidelines from the European Organization for Research and Treatment of Cancer (EORTC) and the National Cancer Institutes of the United States and Canada, entitled Response Evaluation Criteria in Solid Tumors (RECIST) [36]. Those recommendations, published in 2000, describe a method of using medical imaging, particularly the reproducible images obtained with computed tomography (CT) and MRI, to measure the longest diameter of a given target lesion, or the sum of the longest diameters for a set of target lesions, before and after therapy. It also defines lesions considered to be the most difficult to measure and it includes bone lesions especially after chemo-treatments [36,37]. Therefore, we did not measure TV and NV during or after neo-adjuvant chemotherapy because it has already been demonstrated that MRI protocols, including static contrast-enhanced and/or TEP protocols, are unable to accurately assess the tumor response [7,38]. Indeed, a static contrast-enhanced examination does not provide adequate detail regarding the percentage of necrosis in a tumor after treatment [31]. Although an increase of tumor volume suggests a poor response, a decrease of volume do not allow us to distinguish PRs and GRs [39,40]. Diffusion and perfusion sequences can be a surveillance tool for chemotherapeutic response assessment, but not in a predictive way before any treatment [41,42]. However, the degree of tumor necrosis measured by dynamic-enhanced MRI sequences has shown to be an indicator of biologic response and correlates well with histopathologic necrotic percentage [42,43]. In our study, we assessed the necrotic volume at diagnosis before any treatment. Semi-automated determination of tumor volume and necrosis, using MRI, is suggested to be accurate and reproducible [44]. It requires manual contouring, which gives accurate results but is time-consuming depending on tumor characteristics and can be subjective. However, volume assessment in radiology has shown in many studies a high degree of inter-observer reproducibility [44,45]. To diminish the bias, the same MRI system was used for all patients, which limits variation between patients as recommended by Therasse et al. and the calculation was done blindly by two independent medical doctors [36]. Minimal differences were observed between the two calculations (data not shown) making the quality of evidence more convincing and insuring less bias. For the necrotic part assessment, we based our delineation on enhanced images, which allow us to differentiate vascularized tumoral areas (e.g., viable tumor cells, granulated or fibrous tissue) and non-vascularized areas (e.g., liquefaction necrosis). However, these images do not distinguish the viable tumor from immature vascularized granulation tissue, fibrous tissue, neovascularity in necrotic areas and reactive hyperemia [30]. Perfusion sequences would have helped to make this distinction, but from 2008 it was not performed routinely. We helped ourselves by combining T1-weighted with STIR sequences [29] to overpass this difficulty. Overall, the appeal of this technique is to approach, a priori, prognostic factors on routine sequence analysis for a daily use. It allows in this study to establish significant correlation between a worse outcome and a higher NV or NP and to correlate NP to Huvos grades after neoadjuvant chemotherapy. Nevertheless, it encourages a systematization of MRI recommendations across protocols to be able to conduct such evaluation routinely in prospective trials. Therefore, axial post-contrast T1W images and STIR sequences would be mandatory to gather all information to determine in each patient all MRI parameters comprising, notably, NV and NP.

Besides this imaging analysis approaching necrosis/hypoxic parts of the OTS, the proteic biomarker analyses reinforce the idea that hypoxia is an important key in OTS local and distant environments and has a strong impact on OTS outcome. In fact, we demonstrated a significant correlation between hyperexpression of HIF-1α/CAIX and a worse OS and EFS, which is consistent with numerous publications [21,46,47]. An expression of HIF-1α over 7.5% at diagnosis leads to an increase of death risk of more than 20 times and an expression of CAIX over 15% to a 25 times increased risk. This is not surprising, as they are underlining the dominant driving force of hypoxia for OTS progression, chemo-resistance and metastatic ability. Surprisingly, the hyperexpression of pS6-RP (e.g., activated mTor pathway) in more than 35% of OTS cells tend to be correlated with a better outcome (OS and EFS). Those data are conducting to dissociate significantly in statistical analyses the HIF-1α signaling impact in OTS from this frequent mTor involvement. The macrophagic exploration confirmed, as in previous publication in OS2006 cohort [27,48], the significant correlation between CD68+ cells and a worse OS. The CD68+ tumors were then associated with an increased risk of death for the patients of nearly nine times. A significant correlation between GC presence and a worse outcome was also outlined. We were able to combine the three biomarkers CD68+/CD163+/GC+ to determine their impact on patient outcome. This is all in line with previous studies in which the density of M2 TAMs, usually CD163+ cells, seems to be correlated to the tumor proliferation, metastatic process and poor outcome [23,24,25,26]. We did not show that a percentage of mononuclear cells CD163+ superior to 50% was significantly associated with a better OS and EFS, as in the entire OS2006 population [27], but with this significant triple association CD68+/CD163+/GC+ we are opening the path for the previously discussed balance between M1 and M2 cells in OTS and the impact of osteoclastic cells on OTS local spreading and worse outcome. However, our results have identified that TAMs were present in the immune infiltrate in a majority of biopsies, as it has previously been reported [26,27,49]. This immune microenvironment seems to be major in OTS and is intricated significantly with hypoxia (moderate to high correlation between macrophagic biomarkers and hypoxia). In fact, hyperexpression of CD68 was correlating with HIF-1α OTS expression, reinforcing their dual and individual worst prognostic impact. Oppositely, hyperexpression of CD163 was interacting statistically with a good prognosis biomarker that is hyperexpression of pS6-RP. Despite these interesting results tending to define specific signatures combining a volumetric necrosis (NV > 50 cm^3^), HIF-1α/CAIX hyperexpressions in OTS cells and presence of specific macrophages, the limits of our study relied on immunohistochemistry technique itself in this OTS heterogeneous tumor and the small size of the studied population. Nevertheless, we evidenced significant signatures defining macrophage environment, OTS state and prognosis. Those results are opening the way to soon propose a larger multicentric study of this imaging and proteic signature to validate those preliminary results. For this enlarged purpose, we need to gather all patients’ images and paired FFPE samples. Such database on OS2006 protocol is currently built up to afford larger assessments in several ancillary projects, including macrophagic, hypoxic and radiological biomarkers (e.g., the ongoing French BoOST-DataS project (BiOlogical OSTeosarcoma data Sharing)). Our prognostic biomarkers and radiological parameters will also be proposed in prospective therapeutic studies presently opening to increase our expertise on those routine parameters and extend them to complementary biomarkers.

To be able to closely follow OTS patients with those macrophagic and hypoxic features at diagnosis, during treatment and post-therapeutic follow-up, new methods using plasmatic biomarkers can be developed, as in adult cancers [50]. Thus, exosomes have emerged recently as promising non-invasive biomarkers released during hypoxic oncogenesis, which might be quantified in patient plasmas. They usually contain a complex cargo of contents derived from the original cancer cell, including proteins, lipids, mRNA, miRNA or DNA that might be specifically measured [50,51,52]. Thus, in the near future, multiple diagnostic assessments measuring or quantifying HIF-1α, CAIX, mTor or CD68/CD163 balance in tumor specimens and/or plasmatic samples might be proposed during the OTS patient journey to evaluate outcome risks. Furthermore, those targets might be also new innovative therapeutic approaches to potentially combine with chemotherapies.

## 5. Conclusions

The immunohistochemical analyses allow us to significantly link overexpression of hypoxic markers, such as HIF-1α and anhydrase carbonic IX (CAIX), to a worst outcome, while mTor activation was correlated with a better survival. We also evidenced that CD68 positive cells, representative mostly of macrophagic M1 polarization, were associated to those hypoxic biomarkers, HIF-1α and CAIX, and that M2 polarization, mostly related to CD163 positivity, was correlated to mTor activation. These findings, involving clinical, radiological and biology data, allowed to hypothesize a dual signature association ready to use routinely on patient samples in future protocols, where hypoxia and TAMs drive the OTS aggressiveness.

## Figures and Tables

**Table 1:** Hypoxic and macrophagic biomarkers studied by immunohistochemistry and their clinical correlations.

IHC Targets	Positive Cells Median (Range)	Hyper/No Expression	OS Correlation	EFS Correlation	Correlation with Necrotic Percentage	Correlation with CD68+ Cells	Correlation with CD163+ Cells
pS6-RP (*n* = 29)	40 (0–90)	25/4	*p* = 0.0657 HR: 4.533 (0.9063–22.67)	*p* = 0.0564 HR: 4.848 (0.9579–24.53)	Rho: 0.044 *p* = 0.839	Rho: 0.3904 *p* = 0.0441	Rho: 0.6008 *p* = 0.0012
pmTor (*n* = 27)	10 (0–80)	19/8	*p* = 0.5865 HR: 1.208 (0.2405–6.072)	*p* = 0.9989 HR: 1.001 (0.1994–5.027)	Rho: 0.286 *p* = 0.197	Rho: 0.6021 *p* = 0.0014	Rho: 0.7374 *p* < 0.0001
HIF-1α (*n* = 28)	1 (0–80)	14/14	*p* = 0.0029 HR: 22.86 (2.910–179.5)	*p* = 0.0172 HR: 9.676 (1.496–62.59)	Rho: 0.115 *p* = 0.61	Rho: 0.5838 *p* = 0.0017	Rho: 0.3021 *p* = 0.1422
CAIX (*n* = 26)	0 (0–40)	12/14	*p* = 0.0082 HR: 25.34 (2.308–278.1)	*p* = 0.0459 HR: 8.357 (1.04–67.16)	Rho: 0.397 *p* = 0.083	Rho: 0.5255 *p* = 0.0084	Rho: 0.3698 *p* = 0.0824
CD163 (*n* = 26)	20 (0–60)	19/9	*p* = 0.6259 HR: 1.551 (0.2658–9.045)	*p* = 0.4467 HR: 1.989 (0.3384–11.69)	Rho: 0.34 *p* = 0.142	Rho: 0.6927 *p* < 0.0001	
CD68 (*n* = 28)	10 (1–40)	15/13	*p* = 0.0214 HR: 8.947 (1.384–57.83)	*p* = 0.0813 HR: 4.583 (0.8274–25.39)	Rho: 0.307 *p* = 0.164	/	Rho: 0.6927 *p* < 0.0001
CD68+/CD163+ (*n* = 28)	/	16/12	*p* = 0.3526	*p* = 0.6123	/	/	/
CD68+/CD163+/GC+ (*n* = 28)	/	8/20	*p* = 0.0515	*p* = 0.0372	/	/	/
Giant cells (GC) (*n* = 29)	/	9/20	*p* = 0.0882	*p* = 0.0178	/	/	/

SD = standard deviation. HR = Hazard-ratio. 95% CI: 95% Confidence Interval. Bold front = statistically significant (*p* < 0.05 or 95% CI excluding 1). OS = overall survival. EFS = event-free survival. IHC = immunohistochemistry. Rho = correlation coefficient: 0–0.30 = negligible correlation, 0.30–0.50 = low correlation, 0.50–0.70 = moderate correlation, 0.70–0.90 = high correlation and 0.90–1.00 = very high correlation.

## Data Availability

The data presented in this study are available in this article (and Supplementary Material).

## Supplementary Materials

The following supporting information can be downloaded at: https://www.mdpi.com/article/10.3390/cancers14061482/s1, Figure S1: Complementary cross-correlations between hypoxic and macrophagic biomarkers.
