# Supplementary material for: Together Intra-Tumor Hypoxia and Macrophagic Immunity Are Driven Worst Outcome in Pediatric High-Grade Osteosarcomas
Source: Cancers (Basel). 2022 Mar 14;14(6):1482. doi: 10.3390/cancers14061482 (PMC8945994; doi:10.3390/cancers14061482)
Supplement: Supplementary file 1 [file cancers-14-01482-s001.zip › cancers-1633446-supplementary.pdf]

cancers

Article

Together Intra-Tumor Hypoxia and Macrophagic Immunity Are Driven Worst Outcome in Pediatric High-Grade Osteosarcomas

Charlotte Nazon [1], Marina Pierrevelcin [2], Thibault Willaume [3], Benoît Lhermitte [2,4], Noelle Weingertner [4], Antonio Di Marco [5], Laurent Bund [6], Florence Vincent [1], Guillaume Bierry [3], Anne Gomez-Brouchet [7], Françoise Redini [8], Nathalie Gaspar [9,10,11], Monique Dontenwill [2] and Natacha Entz-Werle [1,2,*]

Supplementary Materials:

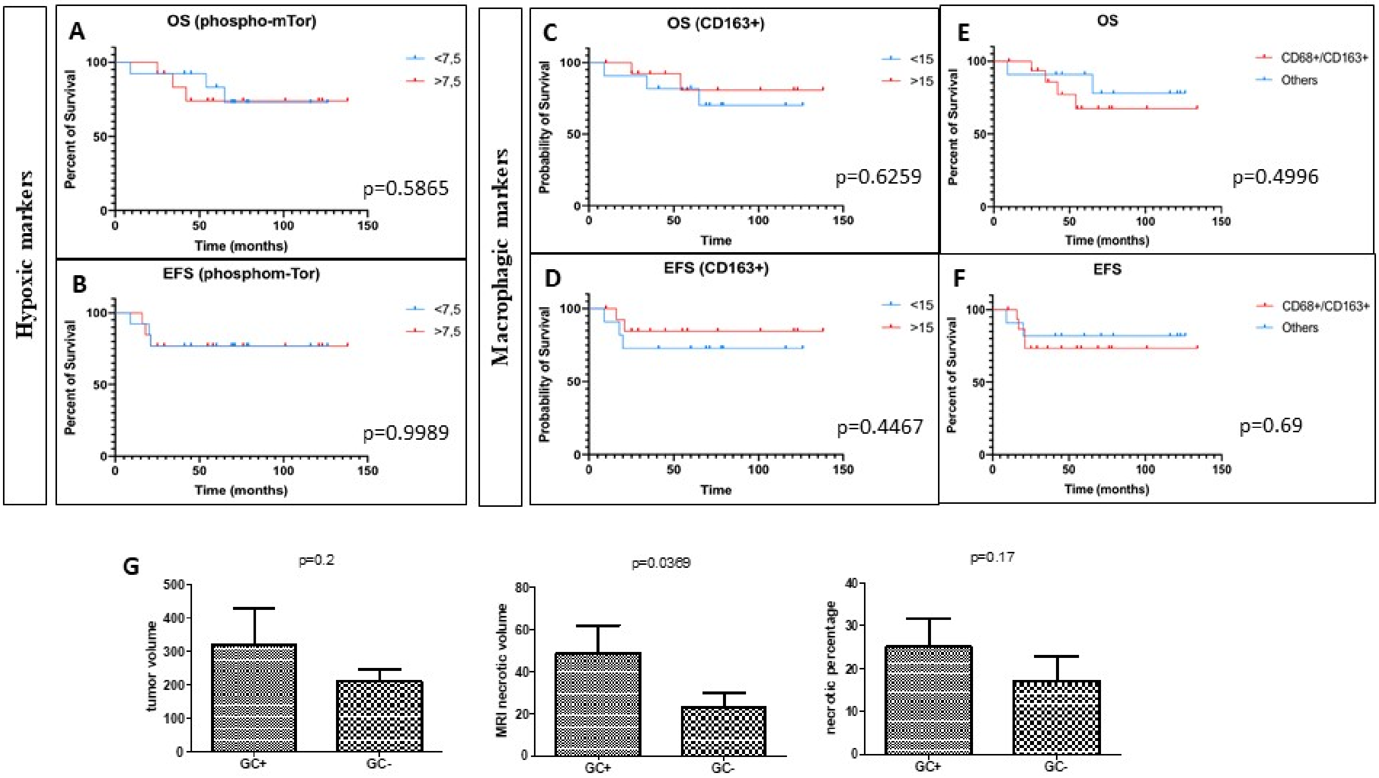

Supplemental Figure S1. Complementary cross-correlations between hypoxic and macrophagic biomarkers. (**A**,**B**) OS and EFS distributions according to the pmTor expression (optimal cutpoint at 7.5%). (**B**–**F**) OS and EFS distributions according to macrophagic markers that are CD163 and the combination of CD163 and CD68. (**G**) Bar graph representations combining MRI data to the presence of giant cells (GC). A significant correlation was evidenced between GC presence and a higher necrotic volume (p=0.0369).
